# Chalazion-mimicked eyelid angiosarcoma in a young Asian with good prognosis: a case report

**DOI:** 10.1186/s12886-023-03262-z

**Published:** 2024-01-02

**Authors:** Gyudeok Hwang, Jong Ok Kim, Ji-Sun Paik, Suk-Woo Yang, Won-Kyung Cho

**Affiliations:** 1 Department of Ophthalmology, Hangil Eye Hospital, #35 Bupyeong‑Daero, 21388 Bupyeong‑Gu, Incheon Republic of Korea; 2 Department of Ophthalmology, Yeouido St. Mary’s Hospital, College of Medicine, The Catholic University of Korea, Seoul, Republic of Korea; 3 Department of Pathology, Daejeon St. Mary’s Hospital, College of Medicine, The Catholic University of Korea, Seoul, Republic of Korea; 4 Department of Ophthalmology, Seoul St. Mary’s Hospital, College of Medicine, The Catholic University of Korea, Seoul, Republic of Korea; 5 Department of Ophthalmology, College of Medicine, Uijeongbu St. Mary’s Hospital, The Catholic University of Korea, #222 Banpo-daero, Seocho-gu, 06591 Seoul, Republic of Korea

**Keywords:** Angiosarcoma, Chalazion, Eyelid, Misdiagnosis, Surgical excision

## Abstract

**Background:**

Angiosarcoma is an extremely rare malignant tumor. So far, only about 42 cases of angiosarcoma involving the eyelids have been reported. Eyelid angiosarcoma occurs more frequently in elderly Caucasian males and is prone to misdiagnosis. We present a case report in a young Asian male patient with eyelid angiosarcoma that was misdiagnosed as a chalazion.

**Case presentation:**

A 46-year-old South Korean male with no underlying disease had a right lower lid mass. The lesion was initially misdiagnosed as a chalazion at a local clinic, but a diagnosis of eyelid angiosarcoma was made after the first biopsy trial. PET-CT was performed to ensure that there was no metastasis in the whole body. Surgical excision with enough surgical margin was used alone for treatment and reconstruction was performed with a tarsoconjunctival advancement flap (modified Hughes procedure), which helped ensure good cosmesis. No recurrence was observed 4 years and 5 months after the surgery.

**Conclusions:**

The current study presents the first case of chalazion-mimicked eyelid angiosarcoma in a young Asian male aged under 50 years. This case shows that even if a benign eyelid disease is suspected in a young patient, an incisional biopsy must be performed to confirm whether the lesion is malignant. Since the prognosis is good for the case of eyelid angiosarcoma, if there is no clear evidence of distal metastasis, surgical resection should be performed with an enough safety margin.

## Background

Angiosarcoma is an extremely rare malignant tumor, accounting for about 0.02% of all cancers [1]. Angiosarcoma can occur in any soft-tissue structure or viscera [2]. So far, only about 42 cases of angiosarcoma involving the eyelids have been reported [3]. Like other head and neck angiosarcomas, the incidence peak of eyelid angiosarcoma is in the eighth decade of life, and it occurs more frequently in elderly Caucasian males. The most common presenting features of eyelid angiosarcoma is a red or bluish maculopapular, which can be easily mistaken for another eyelid disease. Of the 42 eyelid angiosarcoma cases, 19 cases (45.2%) were misdiagnosed as other eyelid disorders [3]. Angiosarcoma that invaded the face and scalp has a very poor prognosis with a 5-year survival rate of 12% [4]. On the other hand, in the case of eyelid angiosarcoma, the 4-year overall survival rate was 48.7%, 4-year event free survival (EFS, survival without recurrence or death) probability was 36.0%. For eyelid angiosarcoma treated with surgical excision, the 4-year EFS probability is 60.6% [3]. In this study, we report a first case of chalazion-mimicked eyelid angiosarcoma in a young Asian man with good prognosis.

### Case presentation

**Fig. 1:**
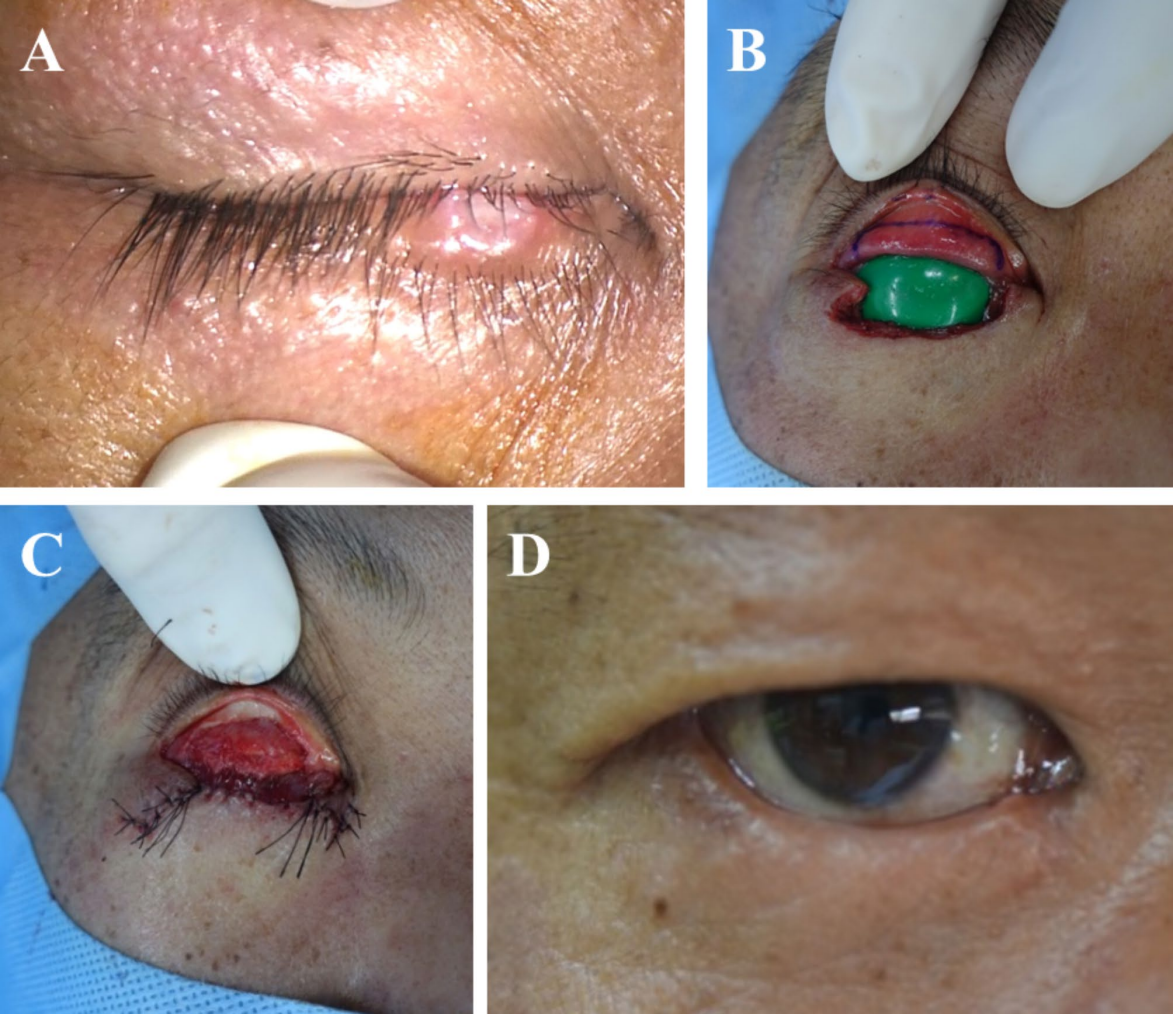
Photographs of our patient. **A**, nodular lesion appearing like a ruptured chalazion in the mid-lower lid margin at the first visit. **B**, after surgical excision with a safety margin of 5 mm and 7 negative frozen biopsies, the incision line was designed at the upper conjunctiva for a tarsoconjunctival advancement flap (modified Hughes procedure) with a green corneal protector. **C**, the tarsoconjunctival advancement flap reconstruction for the posterior lamella and myocutaneous advancement for anterior lamella reconstruction were performed. **D**, no recurrence was observed 4 years and 5 months after the surgery

A 46-year-old South Korean male with no underlying disease had a right lower lid mass and drained it by himself, considering it to be a chalazion. After 20 days, the mass did not improve, so he visited a local ophthalmologist and was referred to our hospital for detection of malignant tumors such as basal cell carcinoma. At the time of the visit, the lesion was a nodule that looked like a ruptured chalazion in the mid-lower lid margin. Definite lid margin destruction, vessel dilatation, and erythematous injection were not observed in the lesion (Fig. 1A).

**Fig. 2:**
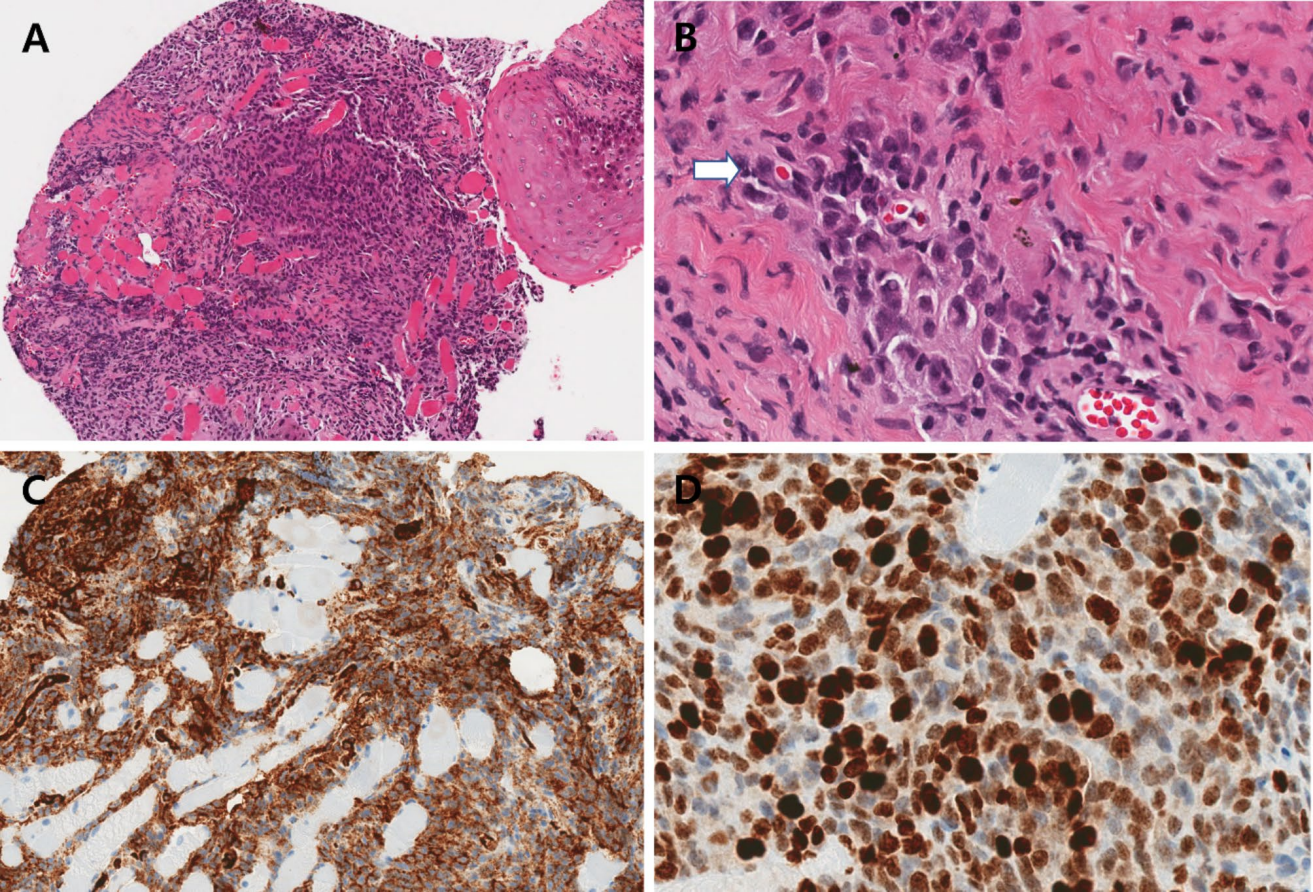
Pathologic findings of angiosarcoma. **A**, atypical endothelial cells showing sheet-like growth and scanty slit-like vascular structures filled with some RBCs and invading skeletal muscle bundles (hematoxylin and eosin, 100x). **B**, atypical endothelial cells lined by vascular structures filled with RBC (arrow)(hematoxylin and eosin, 400x). **C**, positive expression for CD31 (immunohistochemistry, 200x). **D**, high Ki-67 index of 95% (immunohistochemistry, 400x)

Two days after the visit, an incisional biopsy was performed. Atypical cells invading the skeletal muscle bundles were observed in the biopsy specimen. The atypical endothelial cells showed solid and slit-like vascular structures filled with red blood cells. The tumor cells were positive for CD31, factor VIII, and vimentin and had a high Ki-67 index of 95% in the immunohistochemical study (Fig. 2). Vimentin stain showed focal positive and P53 was positive in several cells. Cytokeratin, epithelial membrane antigen (EMA), P63, smooth muscle actin, epithelial membrane antigen, smooth muscle actin, HMB-45, chromogranin, synaptophysin, CD34 and S-100 protein stains were all negative. Taken together, these findings indicated an eyelid angiosarcoma. Positron emission tomography-computed tomography (PET-CT) and enhanced orbital magnetic resonance imaging (MRI) showed no evidence of distant metastasis. Two weeks later, tumor excision with a safety margin of 5 mm and frozen biopsies at seven sites were performed under general anesthesia. The frozen biopsies showed negative margins, and reconstruction of the right lower lid was performed with a tarsoconjunctival advancement flap (modified Hughes procedure) for the posterior lamella and myocutaneous advancement for the anterior lamella (Fig. 1B and C). Six weeks after the surgical excision and lid reconstruction, a split operation between the upper and lower eyelids was performed on the right eye. The patient showed no recurrence 4 years and 5 months after surgery (Fig. 1D).

## Discussion and conclusions

**Fig. 3:**
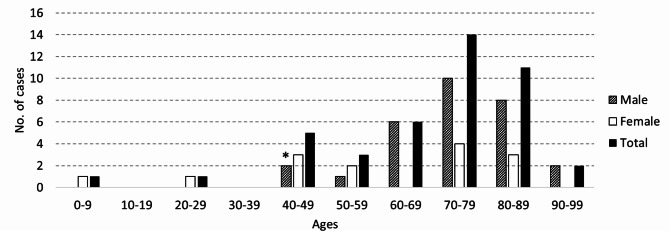
Age and sex distribution of eyelid angiosarcoma. Forty-two cases reported in the paper by Hwang et al. [3] and our current study case were included. * Current study case

The current study presents the first case of chalazion-mimicked eyelid angiosarcoma in a young Asian male aged under 50 years (Fig. 3; Table 1). The lesion was initially misdiagnosed as a chalazion, but a diagnosis of eyelid angiosarcoma was made after the first biopsy trial.

Eyelid angiosarcoma occurs more frequently in people over 70 years of age. So far, only 6 cases have reported in people under the age of 50 (Fig. 3). Eyelid angiosarcoma was most common in Caucasians (40.5%) and found in only 9.5% among Asians [3]. Eyelid angiosarcoma is prone to misdiagnosis. Hwang et al. reported that 19 of the 42 cases (45.2%) were misdiagnosed before the pathologic confirmation of an eyelid angiosarcoma, but none of young patients under the age of 50 was misdiagnosed as another eyelid disease [3].

On the other hand, the chalazion is very common in young adults. The most important feature of chalazion is the slowly growing painless cystic lesions on the eyelid [5]. The most common feature of the eyelid angiosarcoma is a reddish or bluish maculopapular lesion. But it can appear only with a change of color or only the shape changes without color changes [3]. Since the characteristics of eyelid angiosarcoma are not clearly differentiated from other eyelid diseases such as chalazion, malignancy cannot be excluded based on the characteristics of mass-like lesions alone. Bajaj et al. reported that cutaneous angiosarcoma can mimick eczema, rosacea, hematoma, scarring alopecia, sebaceous cyst, rhinophyma, granuloma and keratoacanthoma [6]. Especially, eyelid angiosarcoma was sometimes misdiagnosed as cellulitis, angioedema, hematoma, thyroid eye disease and hemangioendothelioma [3] (Table 1).

The present case shows how important it is to make a definitive diagnosis through biopsy even for lesions that have characteristics of an eyelid disease common to young patients, such as a chalazion.

Angiosarcoma was known to have very poor prognosis. Even a localized angiosarcoma shows a median survival of 7 months [2]. However, the prognosis for eyelid angiosarcomas was better than that of angiosarcomas invading the face and scalp. The median overall survival time was 48 months and the median EFS time was 36 months in eyelid angiosarcoma. Surgical excision was the most important treatment modality to improve the prognosis for eyelid angiosarcoma. The median EFS of the patients who underwent surgical excision was 48 months and the median EFS without surgical excision 12 months [3]. Although the prognosis of the eyelid angiosarcoma is good, cases metastasizing to other organs have a very poor prognosis [3]. Therefore, it is necessary to perform a systemic workup using PET-CT, MRI and CT.

In the current case, surgical excision with enough surgical margin was performed alone for treatment, and the patient showed good prognosis without recurrence for 4 years and 5 months. Frozen biopsy was also performed to make En Bloc excision. PET-CT and enhanced orbital MRI were performed to ensure that there was no metastasis in the whole body. Postoperative radiation therapy or chemotherapy was not performed because the safety margin of surgical excision was wide enough, and there was no sign of distant metastasis.

Hwang et al. said that there were only 4 cases out of 42 cases where clearly stated that frozen biopsy was performed, so the difference in prognosis due to frozen biopsy could not be known. However, the authors believe that En Bloc excision including frozen biopsy must be performed to prevent recurrence and metastasis and obtain a good prognosis.

Pathologically, abnormal, pleomorphic and malignant endothelial cells are the hallmark of angiosarcoma [2]. But, morphological differences from haemangioma, lymphangioma can be subtle [7]. Only with light microscopy, angiosarcoma can be misinterpreted as a benign proliferative or inflammatory lesion [2]. Therefore, immuno-histochemistry (IHC) studies are essential to diagnose eyelid angiosarcoma.

Angiosarcomas typically express endothelial markers including Von Willebrand factor, U europaeus agglutinin 1, CD31 and CD34 [2]. CD31 is more specific, but also expressed in macrophages, histiocytes and plasma cells [7]. Vimentin is a protein that is expressed in mesenchymal cells. The epithelioid angiosarcoma, a highly aggressive and unique morphologic subtype of angiosarcoma is almost always strongly positive with vimentin staining. But, in general, strongly positive vimentin can be interpreted as melanoma, and mesothelioma, renal cell carcinoma, endometrial adenocarcinoma, salivary gland carcinoma, and follicular thyroid carcinoma [8]. Ki-67 index can be used as a diagnostic tool to distinguish between benign and malignant vascular lesions. A higher index of Ki-67 suggests malignant vascular tumors [9]. Although the incidence of P53 mutations in angiosarcoma is actually lower than in other soft tissue sarcomas, P53 mutations may be seen in more than half of angiosarcoma cases [7].

EMA immunoreactivity is reported to be seen in 10% of angiosarcoma, but positivity of EMA or P63 is particularly observed in squamous cell carcinomas [10]. The absence of melanocytic markers such as S-100 can help distinguish angiosarcoma from melanoma [2].

The biopsy specimen of our present study showed atypical cells invading skeletal muscle bundles and atypical endothelial cells showing solid and slit-like vascular structures filled with some RBCs in hematoxylin and eosin (H&E) stain. CD31, vimentin, Ki-67, P53, EMA, P63, smooth muscle actin, CD34 and S-100 stains were performed for IHC studies. The tumor cells were positive for CD31 and focal positive for vimentin and showed a high Ki-67 index of 80% (Fig. 2). P53 was positive in several cells, but EMA, P63, smooth muscle actin, CD34 and S-100 stains were all negative.

According to recent studies, erythroblast transformation specific related gene (ERG) is known to have very high sensitivity and specificity in diagnosing angiosarcoma [11, 12]. If ERG staining had been performed in the present study as well, the probability of a positive result would have been high. However, Miettinen et al. [13] reported that although ERG sensitivity was high at 96%, it was not 100% in angiosarcoma. In addition, ERG shows a strong correlation with CD31, and CD31 is known to be a highly specific marker for angiosarcoma [11, 12]. Additionally, according to McKay and colleges [12], angiosarcoma can be diagnosed straightforwardly if vasoformative elements are observed.

The biopsy specimen of our present study showed vasoformative elements in H&E stain, CD31 positive findings in IHC staining, and no IHC staining findings that could be considered other diseases, so the authors believed that our present case can be diagnosed as angiosarcoma.

Various reconstruction methods were performed after the lesion of the eyelid angiosarcoma was surgically removed [3]. The primary goals of eyelid reconstruction are to protect adequate eyelid function and to achieve acceptable aesthetic appearance [14, 15]. The reconstructive plan will be determined mainly by the defect size, location, and the elasticity of the surrounding tissues, which in turn depend on the patient’s age [15]. The vertical, horizontal, and depth dimensions of the eyelid defect must be determined for defect size evaluation [14]. The close attention should be paid to the integrity of the lacrimal apparatus if the defect involves the medial canthal region [15]. Because the eyelid is a layered bi-lamellar structure, appropriate layered reconstruction is necessary. For anterior lamellar reconstruction, skin graft, flap or advancements can be applied. For posterior lamellar reconstruction, different tarsoconjunctival grafts are recommended depending on the defect range.

In the current case, the eyelid defect involved full thickness 80% of the right lower lid margin after surgical excision. A myocutaneous advancement for the anterior lamella and a modified Hughes procedure for the posterior lamella were performed (Table 2; Fig. 1). A modified Hughes procedure requires covering the visual axis for a certain period of time, and a split operation to separate the upper and lower eyelids is required as a secondary operation. Since the current case is not a ‘last eye’ patient, a modified Hughes procedure could be chosen for reconstruction and helped ensure good cosmesis for a young patient.

In conclusion, even if a benign eyelid disease is suspected in a young patient, an incisional biopsy must be performed to confirm whether the lesion is malignant. Since the prognosis is good for the case of eyelid angiosarcoma, if there is no clear evidence of distal metastasis, surgical resection should be performed with an enough safety margin. IHC studies are essential to diagnose eyelid angiosarcoma.

**Table 1:** Misdiagnosed diseases prior to pathologic confirmation of eyelid angiosarcoma

Misdiagnosis	No. of cases
Cellulitis	8
Angioedema	5
Hematoma	3
Chalazion	2^*^
TED	1
Hemangioendothelioma	1

Forty-two cases reported in the paper by Hwang et al. [3] and our current study case were included

No. = Number; TED = Thyroid eye disease

*Current study case

**Table 2:** Methods used for surgical reconstruction of the defects

Reconstruction method	No. of cases
Forehead flap	4
Free skin graft	4
Split skin graft	2
Eyelid rotation flap	1
Tarsal sliding flap	1
Pedicle flap	1
Cheek rotation flap	1
Modified Tenzel flap	1
Modified Hughes flap	1^*^

Forty-two cases reported in the paper by Hwang et al. [3] and our current study case were included

No. = Number

*Current study case

## Data Availability

All data generated or analyzed during this study are included in this published article.

## List of abbreviations

EFS: event free survival, survival without recurrence or death
EMA: Epithelial membrane antigen
PET-CT: Positron emission tomography-computed tomography
MRI: Magnetic resonance imaging
IHC: immunohistochemistry
